# Supplementary material for: Macrophage migration inhibitory factor is required for NLRP3 inflammasome activation
Source: Nat Commun. 2018 Jun 8;9:2223. doi: 10.1038/s41467-018-04581-2 (PMC5993818; doi:10.1038/s41467-018-04581-2)

Macrophage migration inhibitory factor is required for NLRP3 inflammasome activation

Lang *et. al.*

Supplementary Figures

Compound	Binding Late (RU)	Stability Early (RU)	Stability Late (RU)	Binding to Reference Spot		
COR123625	6.4	0.4	0.0	No		
	R_{50} Method	Steady State Method	Kinetics Method			Predicted Stoichiometry of Complex†
	R_{50} (μM)	K_D (μM)	K_D (μM)	k_a ($10^3 M^{-1} s^{-1}$)	k_d ($^{10-3}$ s^{-1})	Molecules bound/MIF timer
	34 ± 9.9	10 ± 1.4	2.5 ± 1.2	0.6 ± 0.2	1.3 ± 0.3	1:1

† *Assuming 3 molecules of MIF are required to form one binding site.*

Supplementary Table 1. Binding and kinetics of COR123625 to immobilized MIF

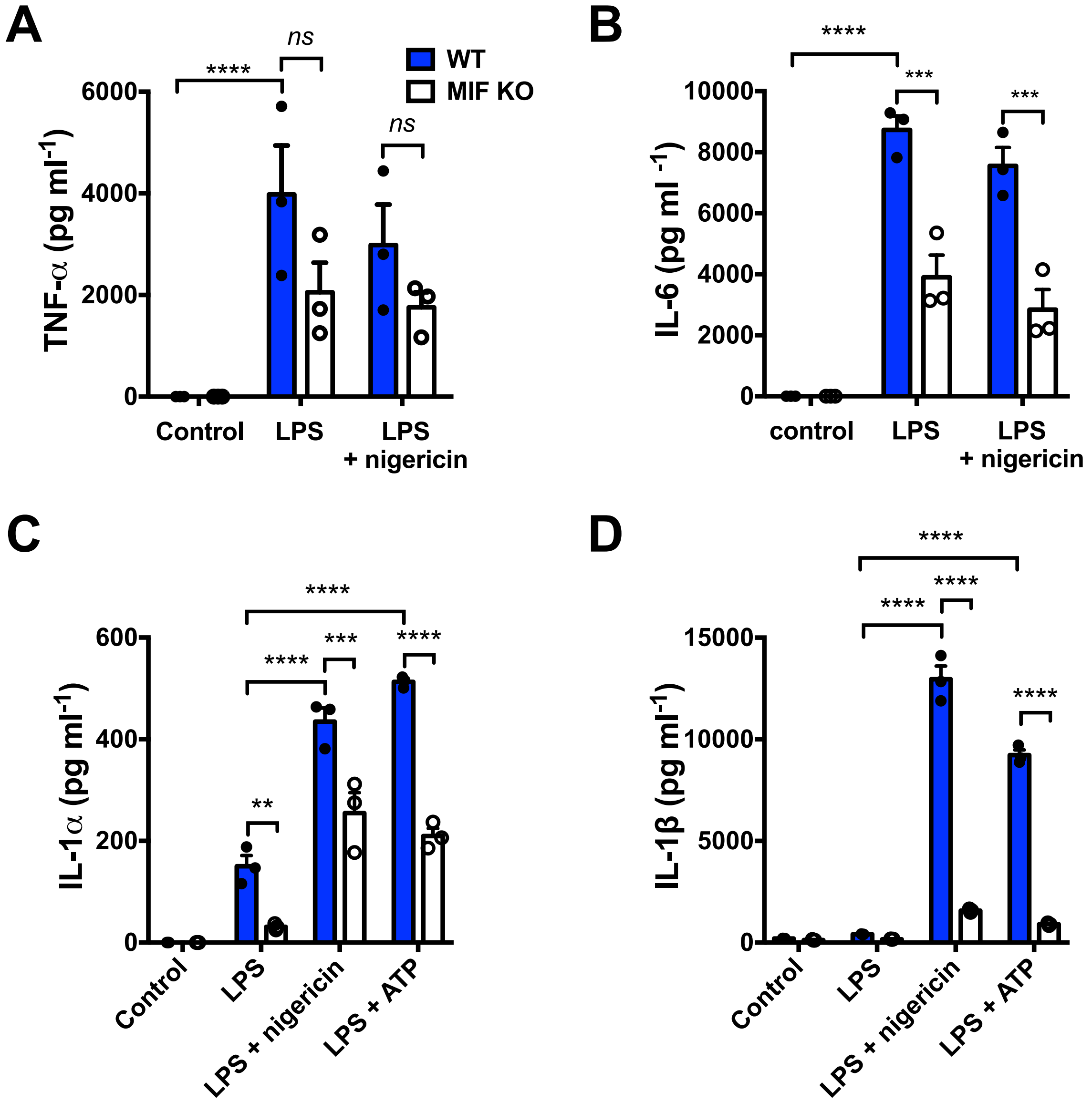

Supplementary Figure 1. MIF is required for release of IL-1 family cytokines. (**A** and **B**) Primary murine WT and *Mif*$^{-/-}$ BMDM were left untreated (control), primed with LPS (10 ng ml^{-1}) or treated with LPS followed by nigericin (5 μM) for 1 h. Levels of **(A)** TNF-α and (**B**) IL-6 in cell culture supernatants were assessed by ELISA. (**C** and **D**) Primary WT BMDC were left untreated, primed with LPS alone (10 ng ml^{-1}), primed with LPS, then treated with COR123625 (50 μM) for 2 h before the addition of nigericin (5 μM) or ATP (10 mM) for 1 h. Levels of **(C)** IL-1α and **(D)** IL-1β in cell culture supernatants were assessed by ELISA. Data are expressed as means ± SEM of 3-4 mice. *$P < 0.05$, **$P < 0.01$, ***$P < 0.005$, ****$P < 0.001$ by one-way ANOVA of variance with a correction provided by the Tukey multiple comparisons test.

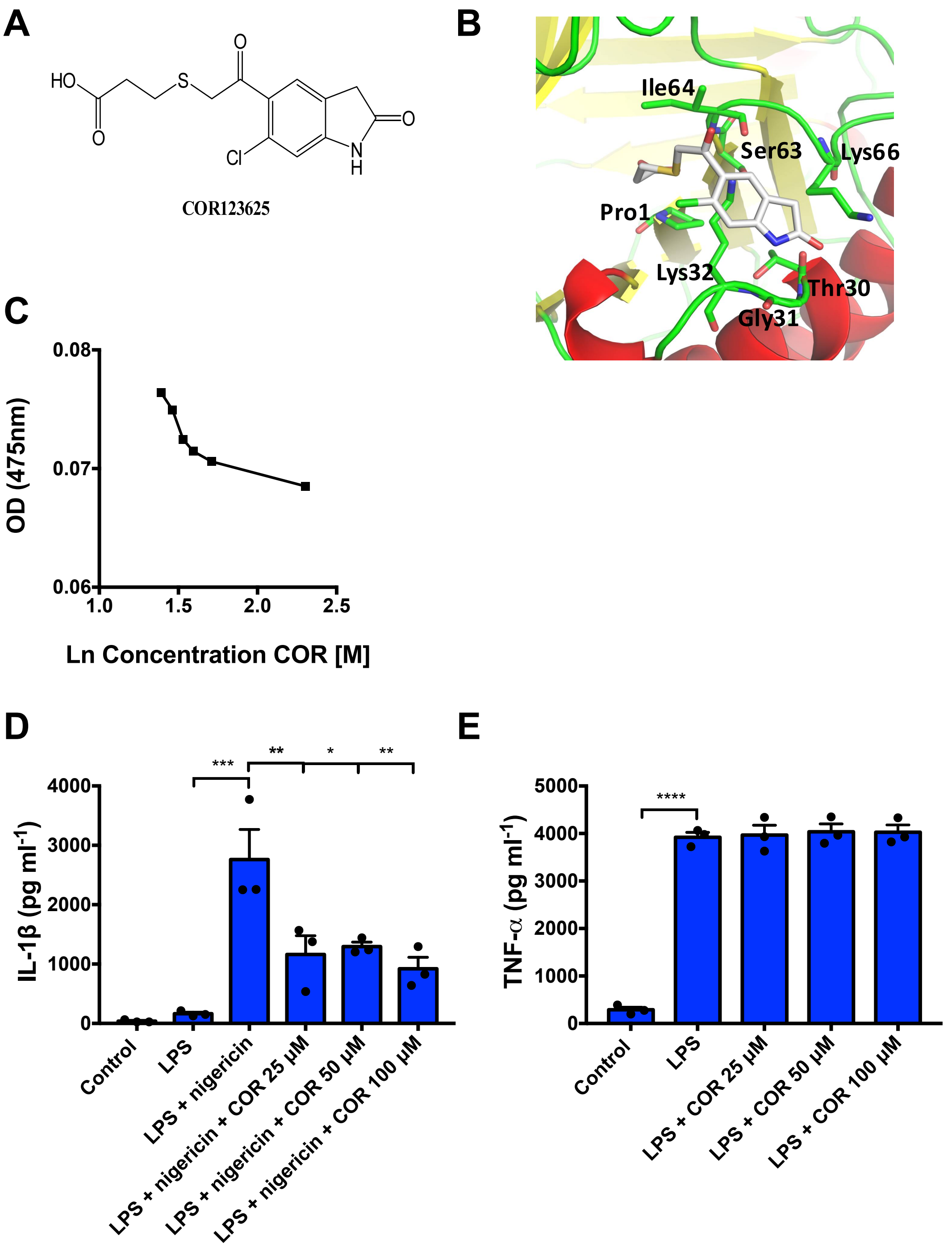

Supplementary Figure 2. COR123625 impairs the biological actions of MIF. (A) Chemical structure of the MIF small molecule inhibitor COR123625. **(B)** The binding position of COR123625 within the tautomerase active site near Pro-1. **(C)** Concentration-dependent inhibition of MIF tautomerase activity by COR123625. (**D**) and (**E**) iBMDM were left untreated, primed with LPS alone (100 ng/ml) for 6 h, or treated with increasing doses of COR123625 (25-100 μM) for 1 h before the addition of LPS for 5 h, followed by nigricin treatment (10 μM) for 1 h. Levels of **(D)** IL-1β or **(E)** TNF-α in cell culture supernatants were assessed by ELISA. Data are expressed as means ± SEM of at least 3 independent experiments. *$P < 0.05$, **$P < 0.01$, ***$P < 0.005$, ****$P < 0.001$ by one-way ANOVA of variance with a correction provided by the Tukey multiple comparisons test.

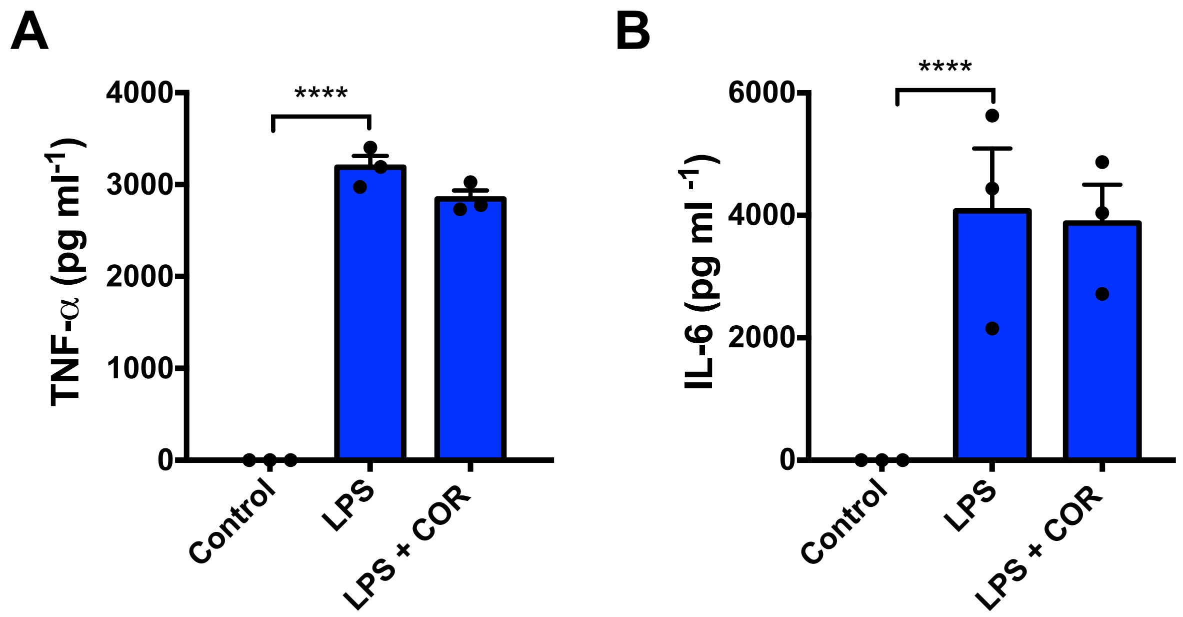

Supplementary Figure 3. Inhibition of MIF does not affect the release of IL-6 or TNF-α. Primary murine WT BMDM were left untreated (control), primed with LPS (10 ng ml^{-1}) for 6 h, or pre-treated COR123625 (50 µM) for 1 h followed by treatment with LPS for 5 h. Levels of **(A)** TNF-α or **(B)** IL-6 in cell culture supernatants were assessed by ELISA. Data are expressed as means ± SEM of 3-4 mice. *$P < 0.05$, **$P < 0.01$, ***$P < 0.005$, ****$P < 0.001$, by one-way ANOVA of variance with a correction provided by the Tukey multiple comparisons test.

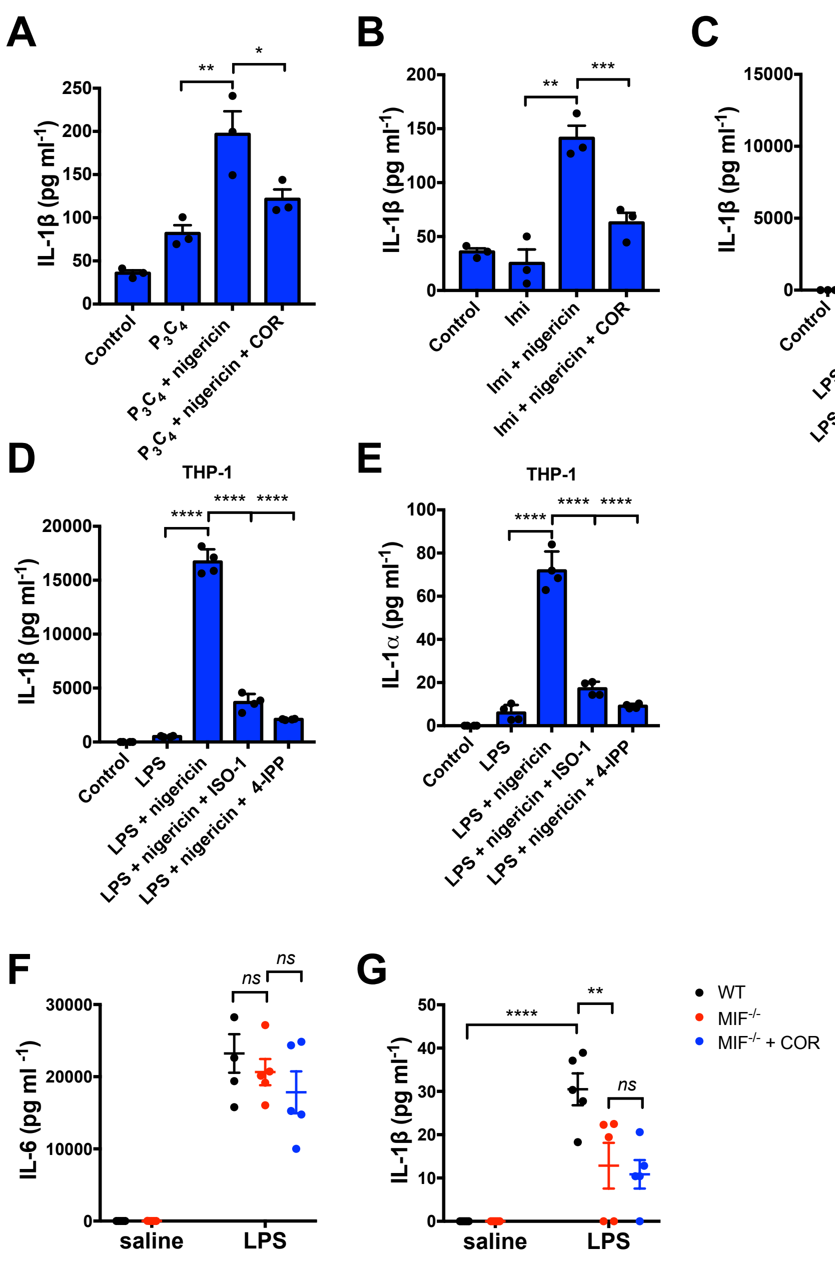

Supplementary Figure 4. Release of IL-1β is inhibited in response to different TLR ligands and different MIF inhibitors. Primary WT BMDM were left untreated, primed with LPS (10 ng ml^{-1}), Imiquimod (Imi; 10 μg ml^{-1}) or Pam$_3$CSK$_4$ (P$_3$C$_4$; 10 ng ml^{-1}) overnight alone, or primed with TLR agonists followed by activation of the inflammasome with nigericin (5 μM) for 1 h, or primed with TLR agonists before treatment with MIF inhibitors COR123625 or 4-IPP (50 μM) for 2 h prior to inflammasome activation with nigericin. **(A-C)** Levels of IL-β in cell culture supernatants were assessed by ELISA. **(D-E)** THP-1 cells were left untreated, primed with LPS (10 ng/ml) overnight, primed with LPS followed by treatment with nigericin (5 μM) for 1 h, or primed with LPS then treated with MIF antagonists ISO-1 (50 μM) or 4-IPP (50 μM) for 2 h prior to inflammasome activation with nigericin. **(F)** and **(G)** WT or MF$^{-/-}$ mice were injected intraperitoneally with vehicle control (saline), LPS alone (2 mg kg^{-1}) or COR123625 (20 mg kg^{-1}) in combination with LPS for 2 h. Serum levels of (**F**) IL-6 and (**G**) IL-1β were measured by ELISA. Data are expressed as mean ± SEM of at least 3 independent experiments. *$P < 0.05$, **$P < 0.01$, ***$P < 0.001$, ****$P < 0.0001$, by one-way ANOVA of variance with a correction provided by the Tukey multiple comparisons test.

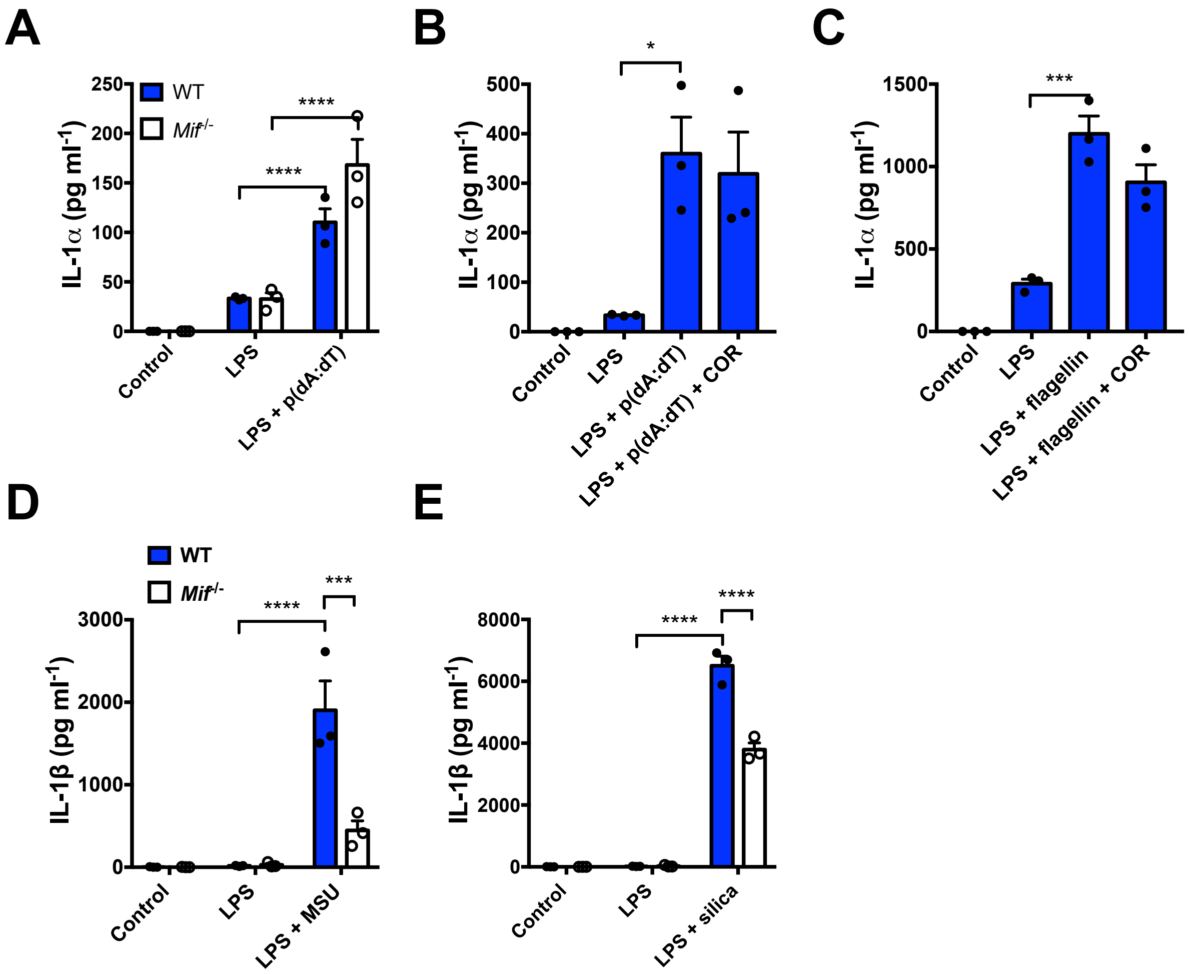

Supplementary Figure 5. NLRP3-dependent release of IL-1α and IL-1β. (**A**) Primary murine WT or *Mif*$^{-/-}$ BMDM were left untreated, primed with LPS alone (10 ng ml^{-1}) or primed with LPS before transfection of poly (dA:dT) (p(dA:dT); 1 µg ml^{-1}) for 5 h. IL-1α release was measured by ELISA. WT BMDM were left untreated, primed with LPS (10 ng ml^{-1}) or primed with LPS before the addition of COR123625 (50 µM) for 2 h before transfection of (**B**) p(dA:dT) (1 µg ml^{-1}) or (**C**) flagellin (250 ng/ml) for 5 h. IL-1α release was measured by ELISA. Primary murine WT and *Mif*$^{-/-}$ BMDM were left untreated (control), primed with LPS (10 ng ml^{-1}) or primed with LPS followed by (**D**) MSU or (**E**) silica for 6 h. IL-1β release was measured by ELISA. Data are expressed as mean ± SEM of at least 3 mice. *$P < 0.05$, ***$P <$ 0.001, ****$P < 0.0001$, by one-way ANOVA of variance with a correction provided by the Tukey multiple comparisons test.

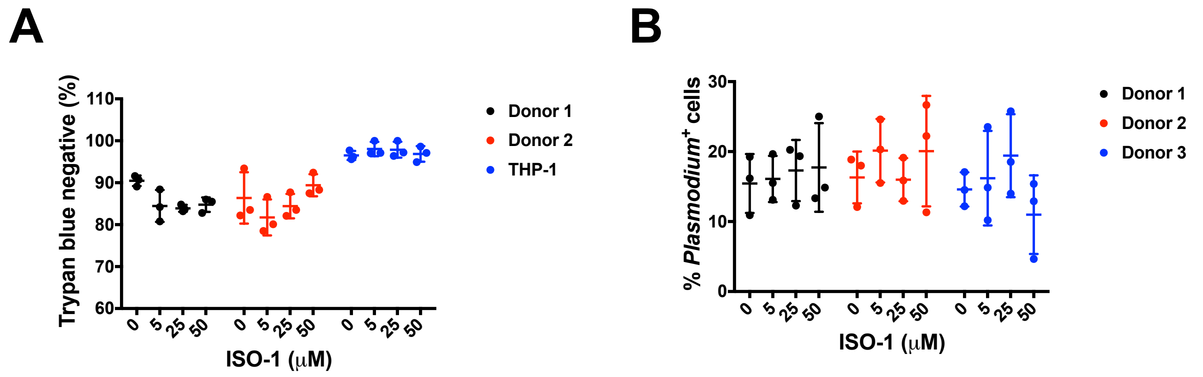

Supplementary Figure 6. MIF inhibition does not induce cell death or prevent phagocytosis of RBC with *Plasmodium falciparum*. (A) Cell viability was assessed by PI staining and data presented as percentage of PI-negative cells. **(B)** *Plasmodium falciparum*-infected RBC were stained with DiffQuik and phagocytosis assessed by light microscopy. Data is presented as the percentage of *Plasmodium* infected cells. Data are expressed as means ± SEM from 3 separate donors.

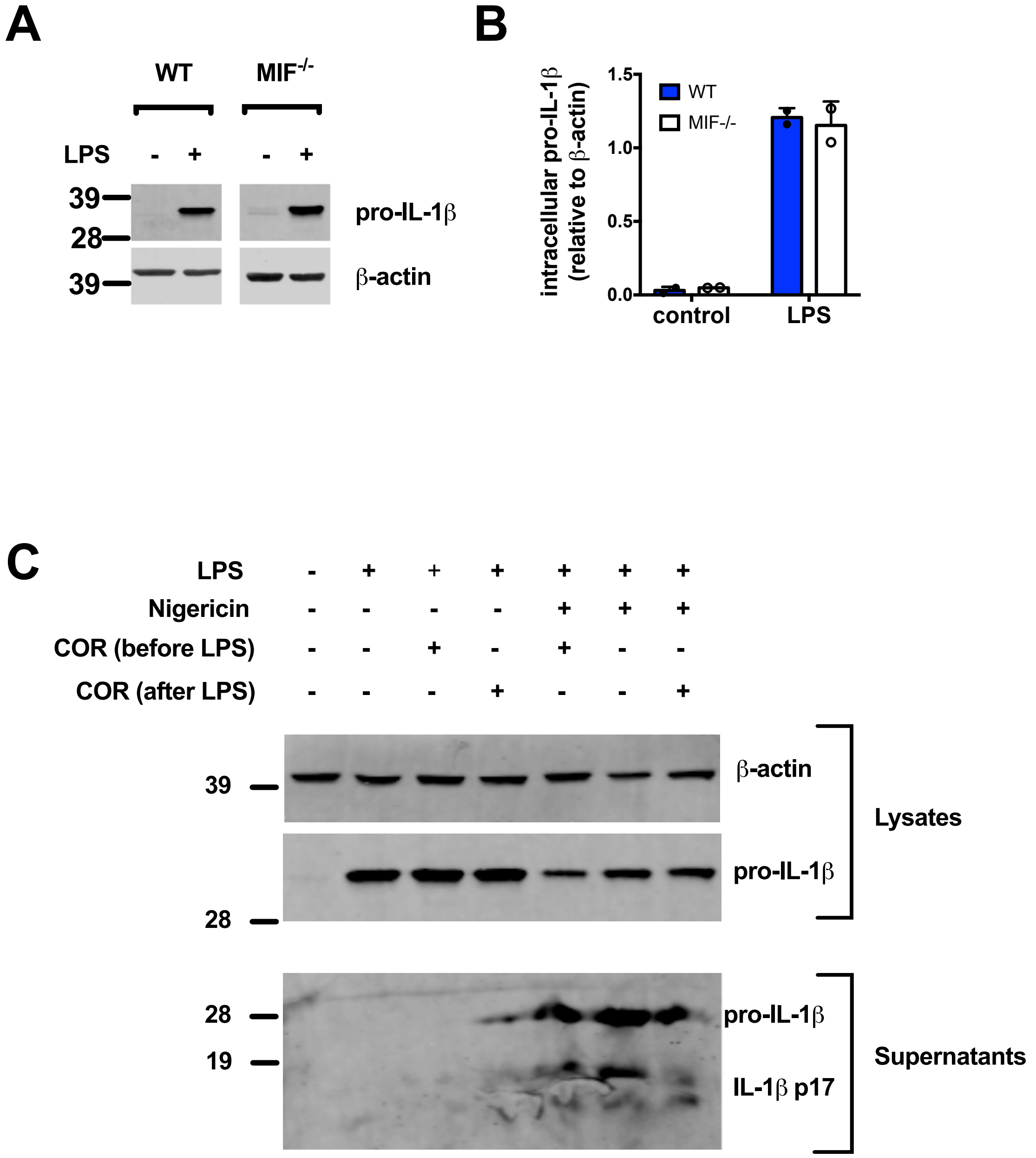

Supplementary Figure 7. Levels of intracellular pro-IL-1β are not affected by MIF. (**A**) Primary WT and MIF$^{-/-}$ BMDM were treated with LPS (10 ng ml^{-1}) for 5 h. Western blot analysis of cellular lysates to assess levels of pro-IL-1β and β-actin was performed. (**B**) Densitometry was used to calculate expression of intracellular proteins shown in (**A**). Expression of pro-IL-1β was normalised to β-actin. n = 2 mice per group. (**C**) Primary murine BMDM were treated with LPS (10 ng ml^{-1}) for 5 h before stimulation with nigericin (5 μM). Cells were treated with COR123625 (50 μM) either 1 h prior to LPS treatment or 1 h prior to nigericin treatment. Western blot analysis of cellular supernatants and lysates to assess levels of pro-IL-1β, mature IL-1β and β-actin was performed.

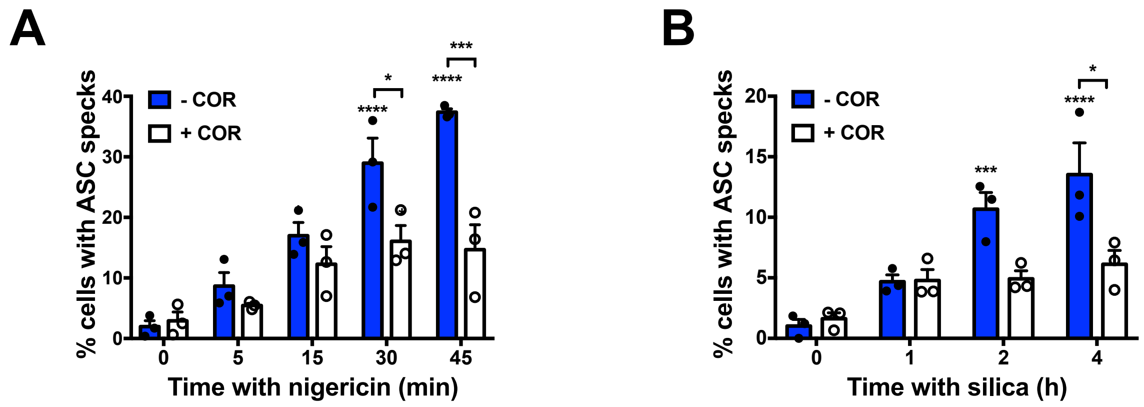

Supplementary Figure 8. Time course for NLRP3 inflammasome activation. ASC-cerulean macrophages were primed with LPS (10 ng mL^{-1}) overnight. Cells were then treated with (**A**) nigericin (10 µM) for 1 h or (**B**) silica (150 µg/ml) for 4 h in the presence or absence of COR123625 (50 µM). Data presented is the percentage of ASC-cerulean cells containing an ASC speck after treatment with nigericin. Data is mean ± SEM of three independent experiments.

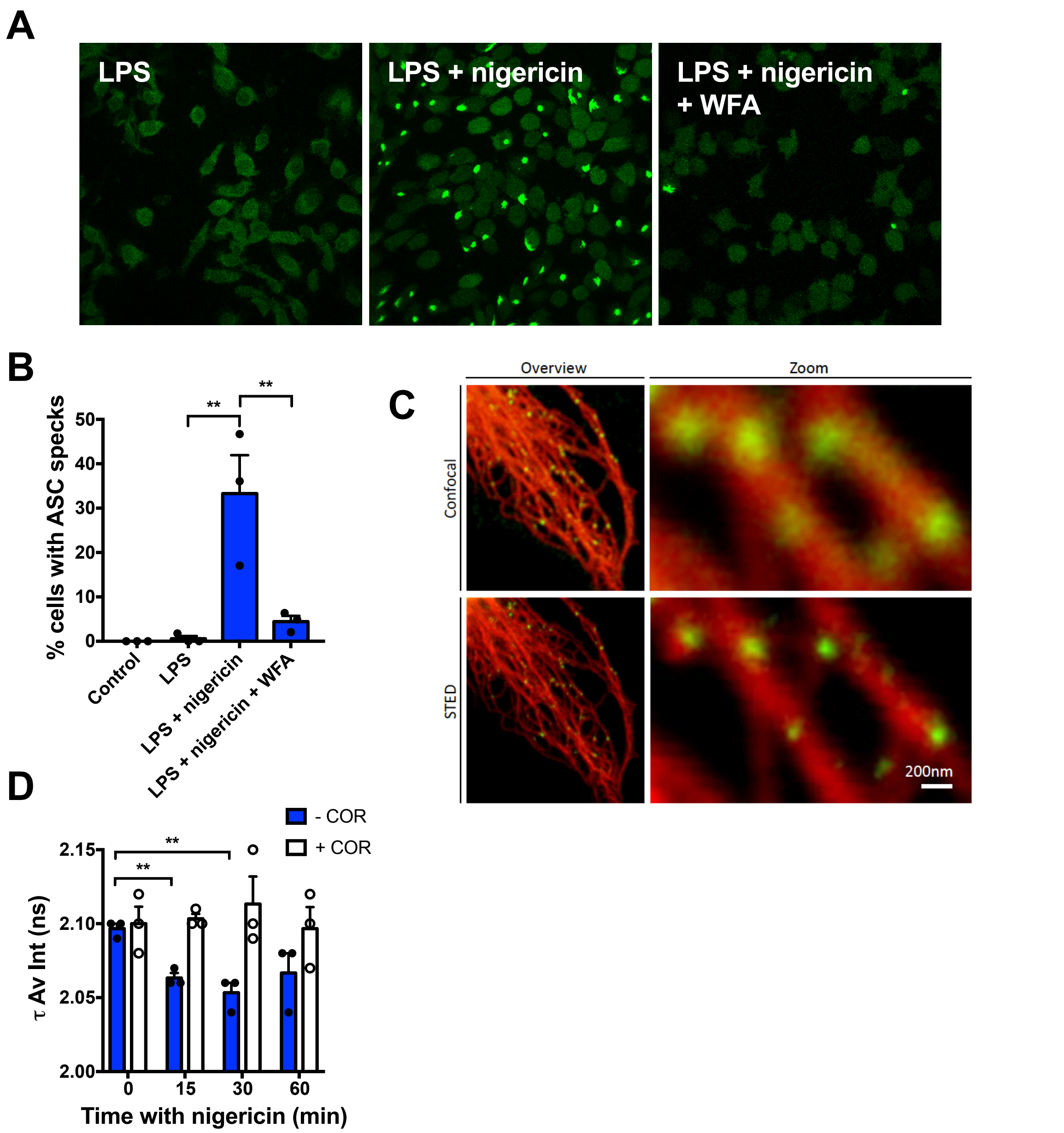

Supplementary Figure 9. MIF and Vimentin are required for NLRP3 activation. (A) ASC-cerulean macrophages were primed with LPS (10 ng/ml) overnight, primed with LPS followed by inflammasome activation with nigericin (5 μM) for 1 h, or primed with LPS followed by treatment with Withaferin A (5 μM) for 2 h prior to nigericin (5 μM) treatment for 1 h. Confocal images shown are representative of at least three independent experiments. **(B)** Data presented is the percentage of ASC-cerulean cells containing an ASC speck. Data shown is the mean ± SEM of three independent experiments. *$P < 0.05$, **$P < 0.01$, ***$P < 0.001$, ****$P < 0.0001$ by one-way ANOVA of variance with a correction provided by the Tukey multiple comparisons test. **(C)** Stimulated emission depletion (STED) Super resolution imaging of vimentin (red) and NLRP3 (green) in WT BMDM treated with LPS and nigericin. (**D**) WT BMDM were primed with LPS (100 ng ml^{-1}) for 5 h, +/- COR123625 (50 μM) for 2 h prior to nigericin (5 μM) treatment for 0-60 min h. Levels of interaction between NLRP3 and Vimentin were assessed by FLIM-FRET. Changes in the amplitude weighted average lifetime (τ Av Amp) of the donor (A488) due to proximity to the acceptor (A568). n = 3 mice, **$P < 0.01$, one-way ANOVA of variance with a correction provided by the Tukey multiple comparisons test.

Supplementary Figure 10: Full length blots for Figure 4D

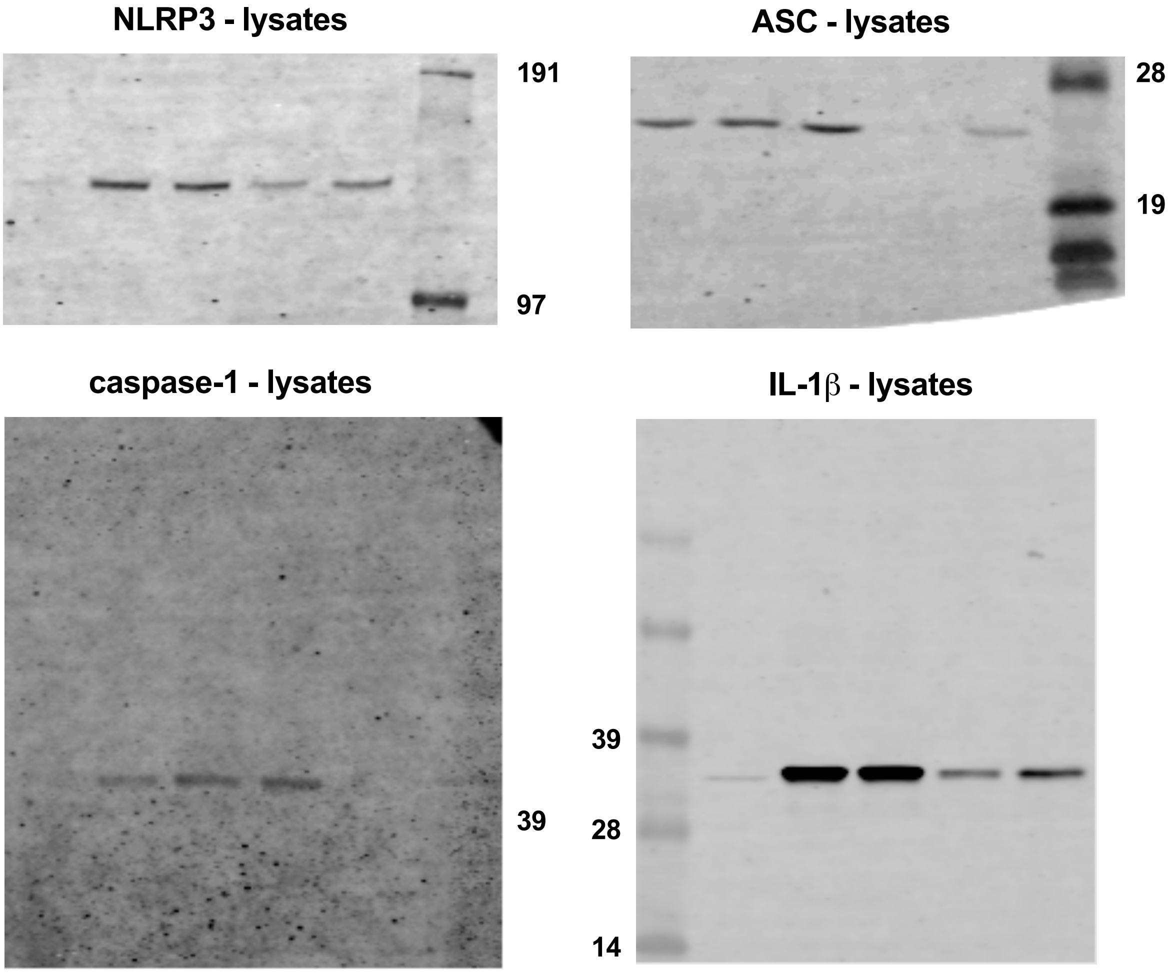

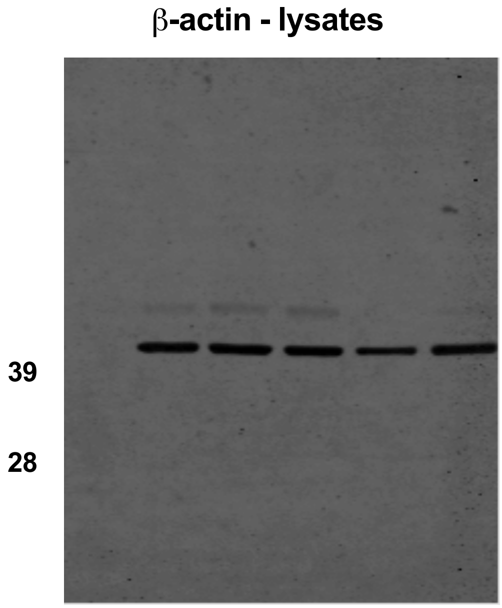

Note: some blots were cut to size to allow simultaneous probing with multiple antibodies.

Supplementary Figure 11: Full length blots for Figure 4D

caspase-1 - lysates

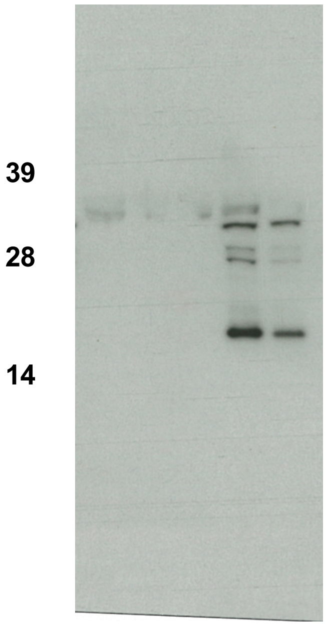

IL-1β - supernatants

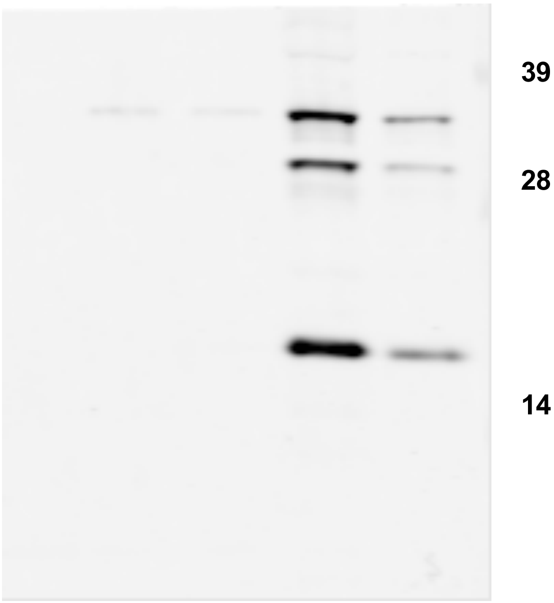

Note: blots were cut above 45kDa

Supplementary Figure 12: Full length blots for Figure 5F

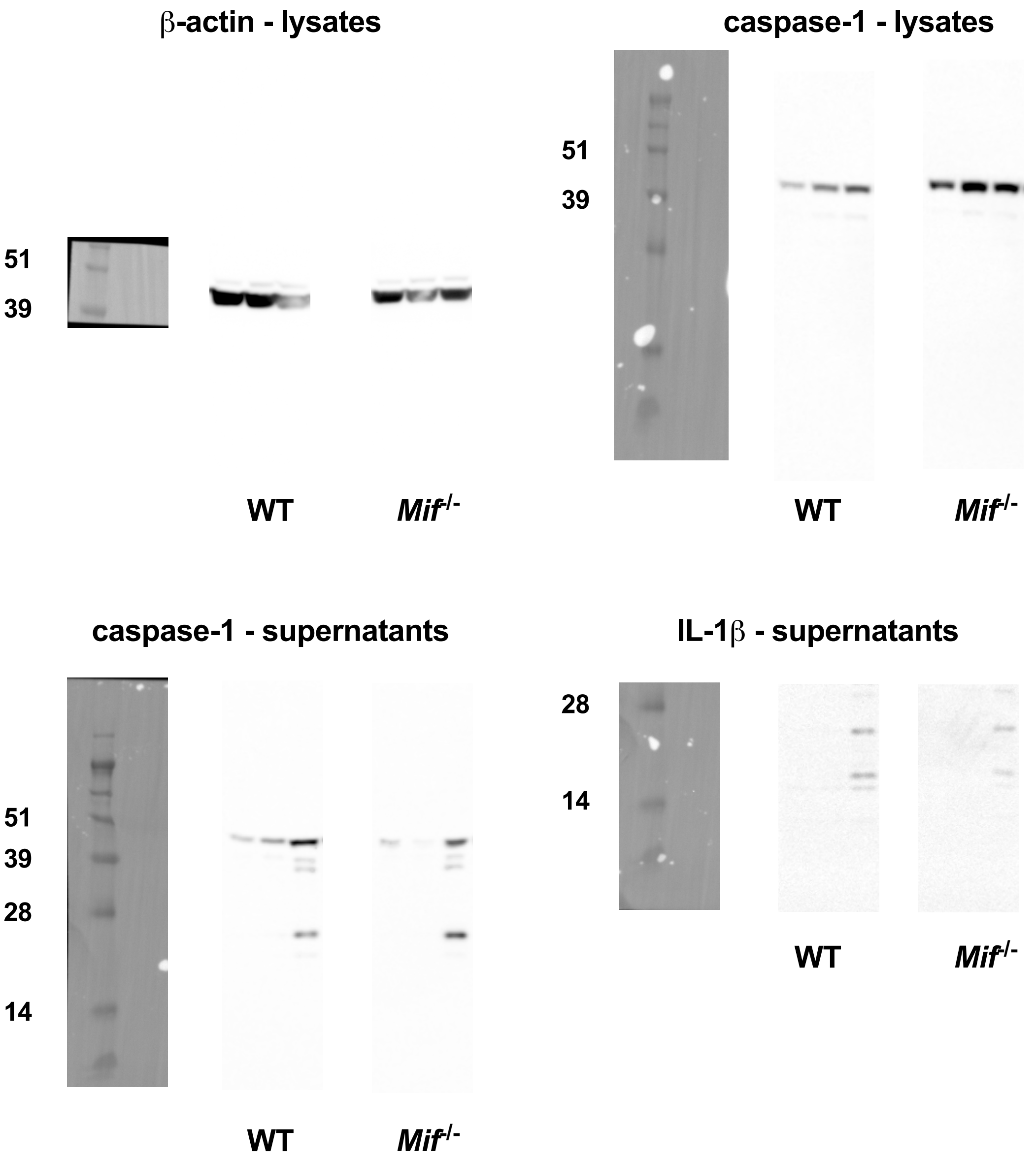

Note: Samples were read on a luminescent image reader and blots were cut for specific sizes. Images on the left are direct photographs of the blot (ladder). Unrelated samples were removed from the blots above.

Supplementary Figure 13: Full length blots for Figure 6A

191

97

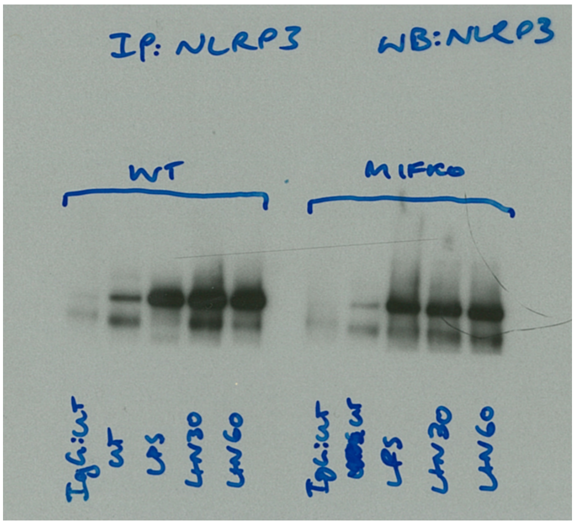

51

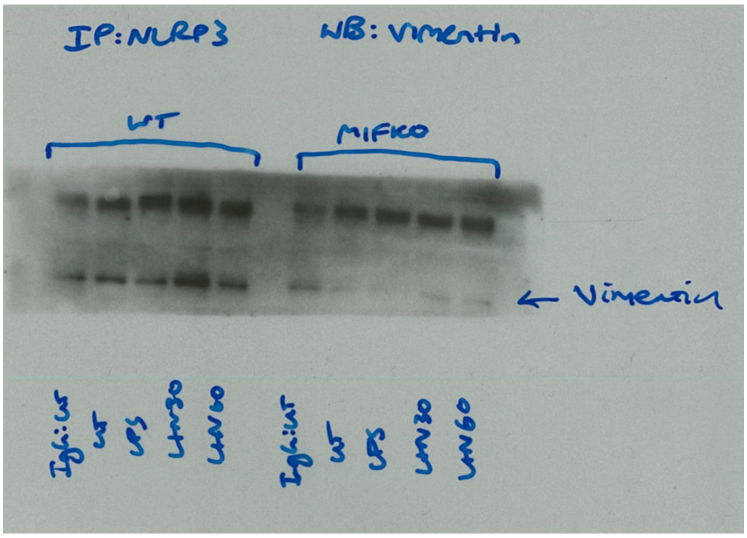

Note: blots were cut to size to allow simultaneous probing with multiple antibodies.

Supplementary Figure 14: Full length blots for Figure 6A

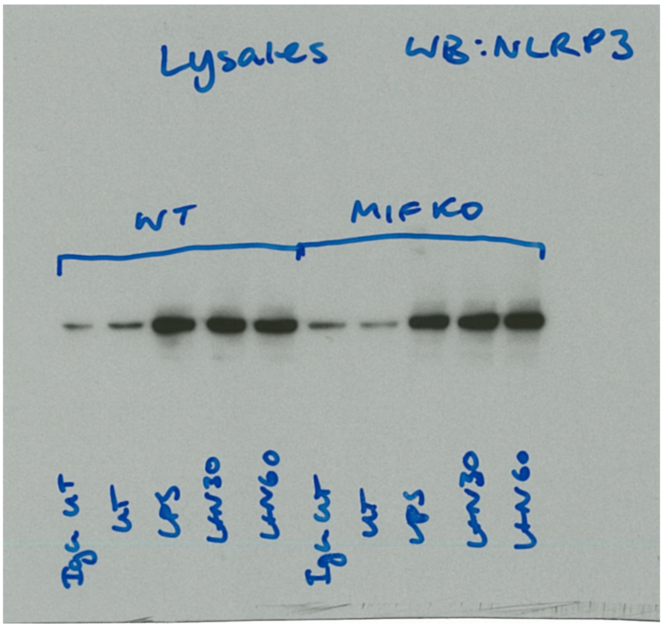

97

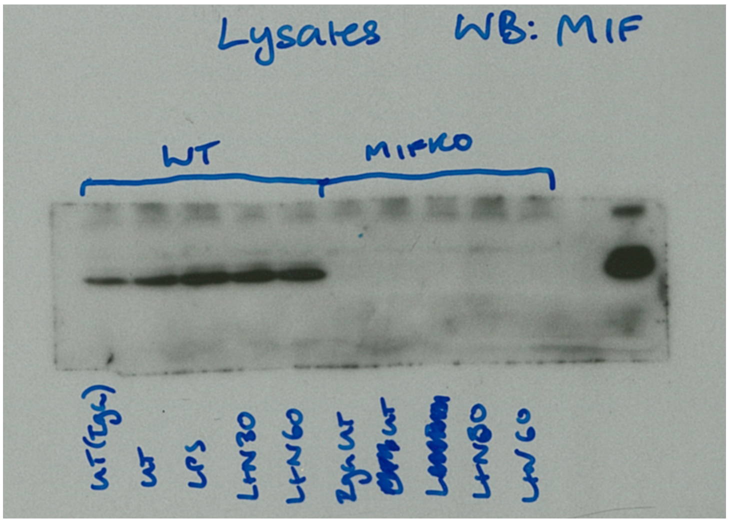

19
14

Supplementary Figure 15: Full length blots for Figure 8A

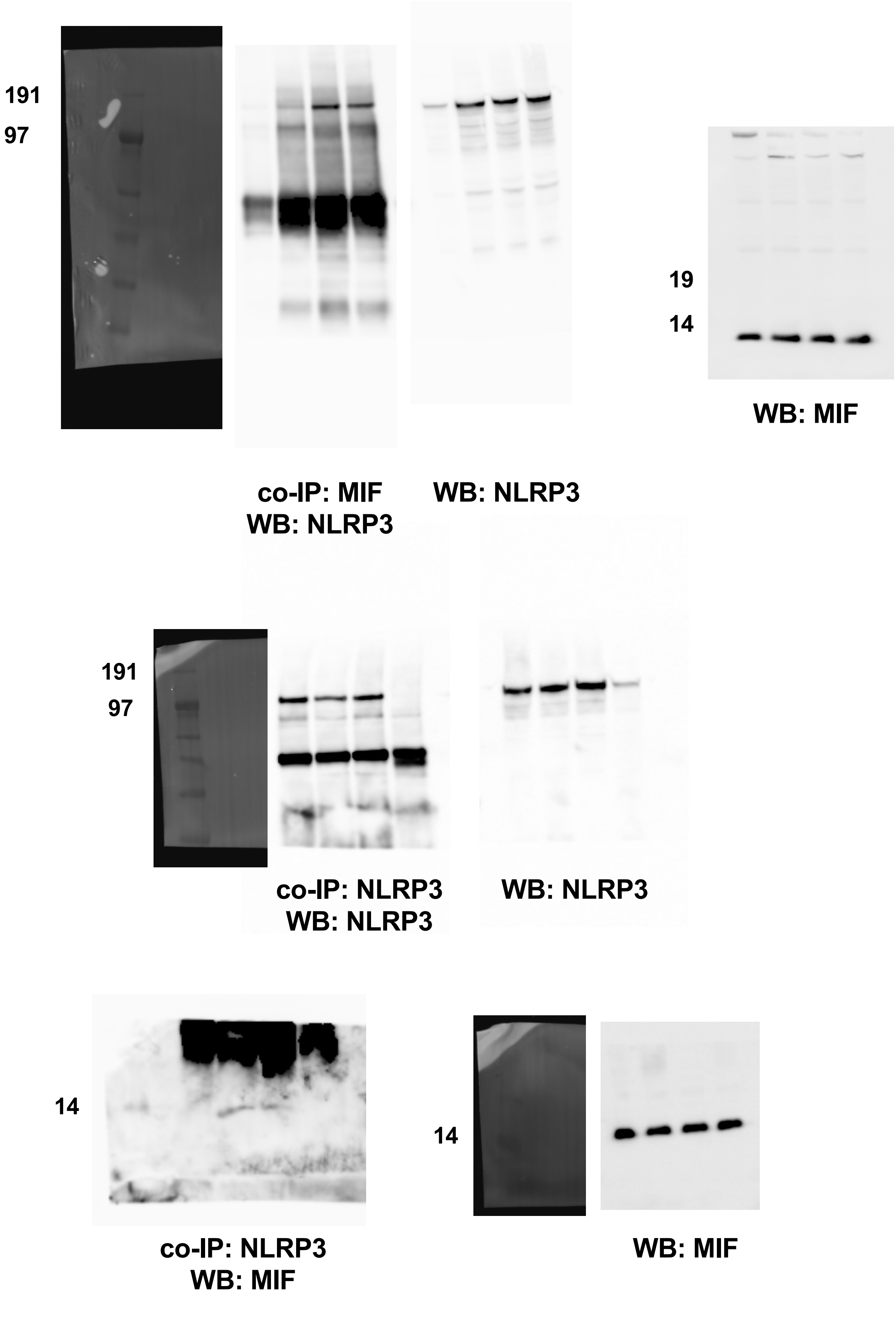

Supplementary Figure 16: Full length blots for Supplementary Figure 7A

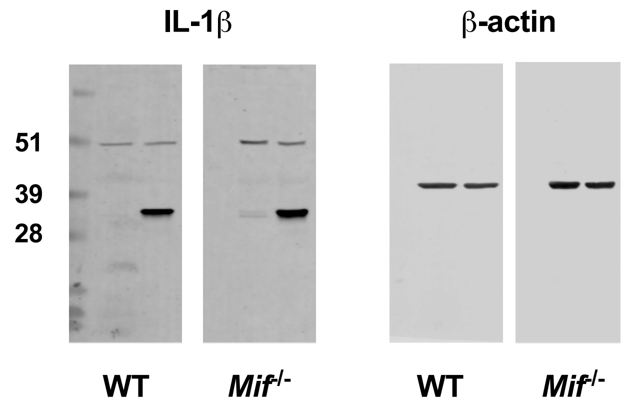

Supplementary Figure 17: Full length blots for Supplementary Figure 7C

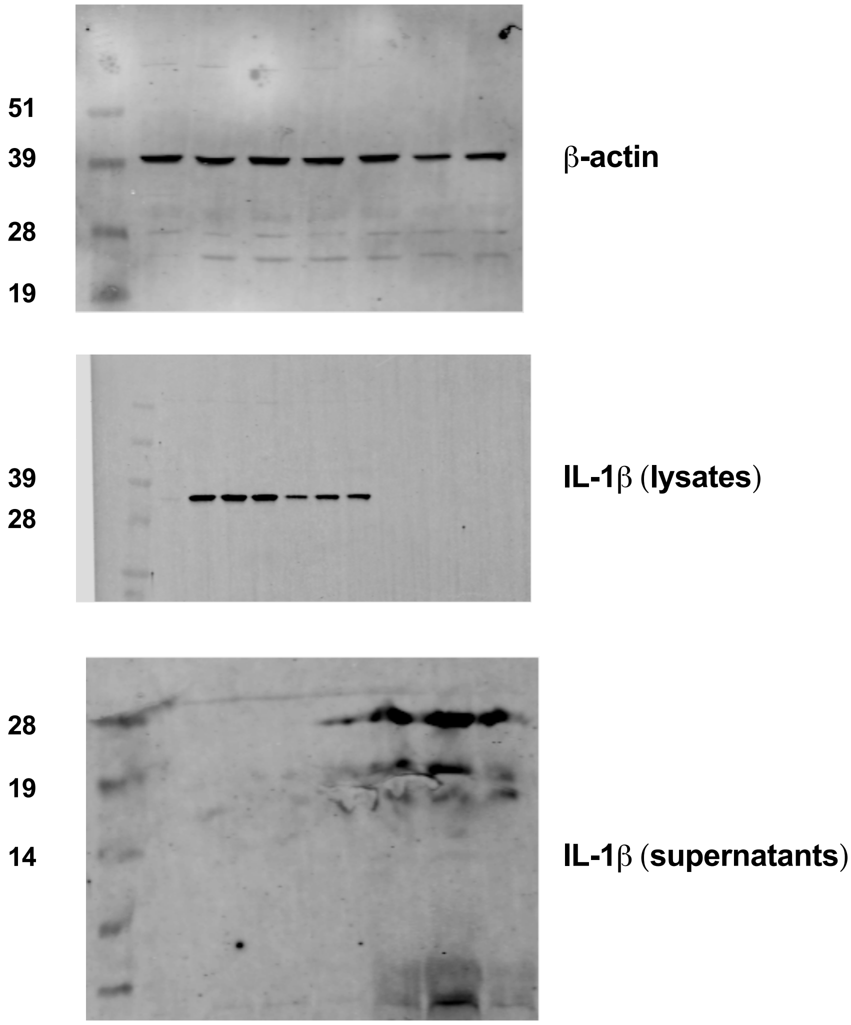

Supplement: Supplementary file 1 — Supplementary Information [file 41467_2018_4581_MOESM1_ESM.pdf]
